# Gender aspects on HIV prevention efforts and participation in HIV vaccine trials among Police officers in Dar es Salaam, Tanzania

**DOI:** 10.1186/s12889-018-5835-5

**Published:** 2018-07-21

**Authors:** Edith A. M. Tarimo, Deodatus C. V. Kakoko, Thecla W. Kohi, Muhammad Bakari, Eric Sandstrom, David Siyame, Fred Mhalu, Asli Kulane

**Affiliations:** 1 Department of Nursing Management, Muhimbili University of Health and Allied Sciences, Dar es Salaam, Tanzania; 2 Department of Behavioural Sciences, Muhimbili University of Health and Allied Sciences, Dar es Salaam, Tanzania; 3 Department of Internal Medicine, Muhimbili University of Health and Allied Sciences, Dar es Salaam, Tanzania; 4 Venhalsan, Karolinska Institutet, Sodersjukhuset AB, Stockholm, Sweden; 5 Health Department Unit, Police Kilwa Road, Dar es Salaam, Tanzania; 6 Department of Microbiology and Immunology, Muhimbili University of Health and Allied Sciences, Dar es Salaam, Tanzania; 7 Karolinska Institutet, Public Health, Stockholm, Sweden

**Keywords:** Gender, HIV prevention, HIV vaccine trial, Police officers, Tanzania

## Abstract

**Background:**

For more than three decades, Human Immunodeficiency Virus (HIV) infection and Acquired Immune Deficiency Syndrome (AIDS) continue to dominate the health agenda. In sub-Saharan African countries, women are at more risk of contracting HIV and AIDS compared with men due to biological, social, economic, socio-economic and cultural factors. Women in the uniformed services may be more vulnerable to HIV/AIDS because of their work context, mobility, age and other factors that expose them to a higher risk of infection than women in the general population. This article describes gender dimensions, motives and challenges towards HIV prevention amongst Police officers (POs) in Dar es Salaam, Tanzania.

**Methods:**

This was a descriptive qualitative study conducted at Police stations in Dar es Salaam, Tanzania. Fifteen in-depth interviews were conducted on POs; seven men, and eight women. Content analysis approach was used to analyze data.

**Results:**

Participants’ self-descriptions shed light on gender differences in relation to self -perceptions, job contexts, sexual relationships and HIV prevention. Both men and women perceived themselves as role models, and believed that the surrounding community perceived the same. Safe sexual behavior appeared crucial to avoid undesirable health outcomes. Risky sexual practices were considered avoidable. Under unavoidable sexual temptations, women in particular would be keen to avoid risky sexual practices. Some participants expressed positive views towards condoms use during extra-marital sexual relationships, while others had negative opinions. Early phases of HIV vaccine trials appeared to gain support from sexual partners. However, condom use during phase I/II HIV vaccine trials was deemed as difficult. Support from the spouse was reported to influence condom use outside the wedlock. However, religious beliefs, socio-cultural issues and individual reasons were perceived as difficulties to promote condoms use.

**Conclusions:**

These findings increase understanding of gender differences and context specific efforts towards HIV prevention. Individuals’ assertiveness against risky sexual practices and the intention to participate in HIV vaccine trials to develop an effective vaccine are worth noting. Nevertheless, uncertainties towards condoms use underscore the importance of condoms’ marketing particularly in extra marital sexual relationships and during early HIV vaccine trials.

## Background

The Joint United Nations program on HIV/AIDS (UNAIDS) statistics show that in 2016, there were an estimated 1.8 million new HIV infections, 36.7 million individuals with prevalent HIV infection, and 1million HIV deaths [1]. Women are regarded as a high risk group for HIV/AIDS worldwide due to gender inequalities and limited or almost no power to exercise their rights regarding safer sex [2]. The consequences of this phenomenon are clearly evidenced by the high figures in the global trends whereby more women and girls get infected with HIV/AIDS annually especially in the low income countries. Similarly, in Tanzania, the HIV epidemic continues to disproportionately affect women and girls. HIV prevalence is higher among women (6.2%) than among men (3.8%) [3]. The drivers of the epidemic among others are unprotected sexual behavior, concurrent sexual partnerships, and inadequate comprehensive knowledge of HIV transmission and prevention. Contextual factors such as poverty and transactional sex with increasing numbers of commercial sex workers, men's irresponsible sexual behaviour, as well as economic and political gender inequalities including violence against women had been found to greatly contribute to the epidemic in Tanzania [3]. The Government of Tanzania in collaboration with other stakeholders is continuously investing in HIV prevention, AIDS care and antiretroviral treatment to save lives of her people. Consequently, HIV prevention remains on the national health agenda. One of the strategies adopted is investment in condom marketing to prevent sexually transmitted infections (STIs), including HIV. Additionally, various groups of individuals, including uniformed officers have been involved in HIV prevention efforts through increasing their awareness to the disease and other measures. Of particular importance is the fact that since 1994, Police officers (POs) in Dar es Salaam, Tanzania have been involved in HIV studies including participating in early phases of HIV candidate vaccine clinical trials.

A previous prospective cohort study was conducted at all 32 police stations located in urban and peri-urban Dar es Salaam, Tanzania revealed that the prevalence of HIV infection was almost the same as the national prevalence [4]. A follow-up quantitative cross sectional study that was carried out in 23 out of 32 police stations that serve both urban and peri-urban areas of Dar es Salaam region indicated that only 54% of POs used condoms during extra marital sexual relationships [5]. A further follow up explorative qualitative study conducted in eight out of 32 police stations in Dar es salaam region, revealed that some POs were hesitant to take part in preventive HIV vaccine trials because of fear of adverse effects in their reproductive biology [6]. Another exploratory qualitative study was conducted among members of the police force in Dar es Salaam using a sub-group of POs; these POs signed up for an HIV and AIDS education workshop illustrated how they viewed the problem of HIV and AIDS, particularly the issue of unsafe sexual practices. The POs reported widespread risky sexual practices both in work place and within the community [7]. Additionally, during Focus Group Discussions (FGDs) of 66 participants, the POs revealed gender power imbalances in sexual negotiations and they reported issues of sexual bribes with job promotions [7]. From this background, it was deemed important to conduct in-depth interviews to understand how individual POs reasoned around HIV prevention including gender issues, condom use and participation in preventive HIV vaccine trials. This paper analyzes gender dimensions, motives and difficulties towards HIV prevention amongst POs in Dar es Salaam, Tanzania.

## Methods

### Design, setting and population

This was a descriptive qualitative study design which builds on a series of socio-behavioral studies, which were conducted in an open cohort of POs between 2007 and 2012 [4–8]. In brief, this was a part of HIV vaccine trial studies where rapport was already created in initial studies. Thus entry to the police stations was smooth through the collaborators, and the awareness about ongoing research projects amongst higher authority personnel. The police authority and individual officers were assured of voluntary participation in HIV/AIDS research projects. For this particular study, participants were from 14 different stations. The stations were well coordinated in terms of leadership and accessibility.

Therefore, the study was conducted at Police stations in Dar es Salaam, Tanzania from July to September 2007. During the study period, the Police force in Dar es Salaam had 32 police stations scattered in urban and peri-urban areas of which a few stations were a short distances apart [5]. Nevertheless, all police stations were well coordinated and accessible through private and public transport. Dar es Salaam region then had approximately 6,000 POs. From the previous study, women formed 20% [5]. High and low- ranking Police officers, both men and women constituted the study population. Between 2005 and 2006, this population received sensitization workshops in preparation of early phase I/II HIV vaccine trials [5].The workshops were conducted during Barazas (weekly meetings at stations) which were designed to inform the POs about HIV/AIDS and intention of conducting HIV vaccine trials.

### Sampling and sample size

Purposeful sampling using pre-determined criteria was used to recruit potential participants. The sampling was conducted through collaborators who provided the list of available officers at stations. A list of high ranking and low ranking officers who did not take part in the previous study [7] were invited to participate in interviews. The authors reviewed the list of those who met the inclusion criteria and available for an interview. There was no any bias in selecting participants for the study. Thus authors did not purposively select people having extra marital sex because the potential participants were not judged according to marital status.

The sample was pre-determined in the sense that level of low and high ranking and gender differences were taken into consideration during recruitment. In addition, the sample size was determined by principles of data saturation. Hence fifteen in-depth interviews were conducted involving seven men and eight women. Despite the fact that we based on the pre-determined sample, we reached saturation because the information became repetitive and no new information was expected to be gained by interviewing more participants.

### Data collection

Following a brief background about the previous study findings, the authors (EAMT and AK) interchangeably interviewed the participants after consenting. They used an interview guide comprising of the following themes:

1. *Gender dynamics in the Police Force; whose probes were:*
  - If you are at the same level (education, experience/rank etc) with a man/woman colleague, what efforts do you need to make sure that you get a promotion?
  - If a man/woman colleague puts pressure on you for sex, e.g. giving money for no apparent reason, invitation for lunch, dinner or other favors, what will you do? How can you influence the situation?
  - How can men and women influence condom use to avoid pregnancy outside wedlock?
2. *HIV prevention efforts among Police Officers, whose probes were*:
  - Can you give suggestions on how condom use can be promoted in more effectively?
  - Police Officers complain that condom use does not bring pleasure. How do you promote condom use in HIV prevention?
  - Why isn’t it possible for couples to influence each other on condom use during extra marital relationships?
  - How can you decide over your sexuality?
  - What would you do if your casual partner demands unprotected sex?
3. *HIV vaccine trial among POs; whose probe was:*
  - If you were to participate in an HIV vaccine trial, what could be the effect on your sexual life?
  - If you were to participate in HIV vaccine trials, what could be the implications of using condoms?

These questions emerged from a previous study [7] in the sense that we planned to seek deeper understanding about gender and sexual relationships and HIV / AIDS issues among POs. Thus, this approach might have influenced the construction of the questions. All interviews took place in privacy in venues of the participants’ preference at the working police stations. One participant insisted to take part in the study; however, she did not prefer audio-recording despite assurance of confidentiality. She reasoned that other people could take note of her voice in case of audio-recorder misplacement. Therefore, fourteen of the 15 interviews were audio-recorded as one of participants declined to be recorded and that decision was respected as per consent. Each of interviews took between 26 and 65 minutes.

### Data analysis

Data was analyzed qualitatively using a content analysis approach as suggested by Granheim [9]. The audio-recorded data were transcribed verbatim. The last author, AK, reviewed 25% of the transcripts to gain a sense of what the informants communicated. EAMT and the second author, DCVK independently coded all the transcripts. Analysis of the two independent authors was then merged. Preliminary categories were constructed and discussed between the two authors. Discrepancies including omissions of important raw data were identified, discussed, reviewed and included in the analysis upon consensus. Categories were revised and re-constructed from the revised codes. Two categories were formed: Gender, sexual relations and condoms use among POs and Perceived readiness to enroll in early HIV vaccine trials among POs. Direct quotes were added to reflect the informants’ voices in the article.

## Results

### Socio-demographic characteristics

The informants consisted of seven men and eight women. The majority was aged 40 years or above, married, and educated up to 4 years of secondary education. Almost half were senior POs such as Inspectors and Superintendent of Police (Table 1).

**Table 1:** Socio-demographic characteristics of study participants

SN	Sex	Age	Education	Marital status	Police Rank
1	M	50	4 years secondary school	Married	Inspector [NSP] (High ranking)
2	M	49	7 years primary school	Married	Staff sergeant [SGT] (Low ranking)
3	M	32	4 years secondary school	Married	Police Constable [PC] (Low ranking)
4	M	42	4 years secondary school	Married	Sergeant [SGT] (Low ranking)
5	F	42	7 years primary school	Not married	Police Corporal [CPL] (Low ranking)
6	M	38	7 years primary school	Married	Police Corporal [CPL] (Low ranking)
7	F	43	4 years secondary school	Married	Police Corporal [CPL] (Low ranking)
8	F	43	7 years primary school	Married	Police Corporal [CPL] (Low ranking)
9	F	24	4 years secondary school	Married	Police Corporal [CPL] (Low ranking)
10	F	40	University graduate	Married	Inspector [NSP] (High ranking)
11	M	54	4 years secondary school	Married	Inspector [NSP] (High ranking)
12	F	55	4 years secondary school	Separated	Assistant Superintendent of Police [ASP], (High ranking)
13	F	54	4 years secondary school	Widow	Superintendent of Police [SP], (High ranking)
14	M	44	6 years secondary school	Married	Inspector [NSP] (High ranking)
15	F	52	4 years secondary school	Married	Superintendent of Police [SP], (High ranking)

### Categories and sub-categories

The first category, ‘*Gender, sexual relations and condoms use among POs’* provides insights about perceptions, distribution of roles, and sexual relationships among participants. The second category, ‘*Perceived readiness to enrol in early HIV vaccine trials among POs*’, analyzes readiness and the foreseen obstacles towards participating in HIV vaccine trials. Each category is supported by sub-categories as presented in the following sections (Table 2).

**Table 2:** Categories and sub-categories

Categories	Sub-categories
Gender, sexual relations and condoms use among POs	Perceptions of the positive image of POs
	Gender roles in the context of Police profession
	Gender and leadership
	Gender based nepotism in Police profession
	Dealing with risky sexual temptations
	Emphasis on protected sex
	Unsure of condom use among sexual partners
Perceived readiness to enrol in early HIV vaccine trials among POs	Partner involvement in HIV vaccine trials
	Obstacles towards participation in HIV vaccine trials

### CATEGORY 1: Gender, sexual relations and condoms use among POs

#### Perceptions of the positive image of POs

Participants felt proud of themselves for being POs. Both men and women believed themselves to be possessing talents and capacity to lead others. Although they observed that duties of POs resemble duties of other professions in terms of responsibility, they believed that they were role models and that the surrounding community perceived the same. They perceived that their daily duties and responsibilities influence civilians to be law abiding citizens. They said they were obliged to practice loyalty, provide constant support to crime suspects and maintain trust towards both civilians and foreigners. Most participants were of the opinion that the role of POs is all about being a good example as a ‘mirror’ that reflects the wisdom of the Police force in the society. They said:

*We* [Police officers] *are the mirror of the society; everybody recognizes that; even Mzungu* [a white skinned man from abroad] *will approach a Police officer for help… I am proud of working as a Police Officer, I feel safe including my family…* (High ranking, 50 years old married man).

Another participant emphasised on the influence of appearance and readiness to help others:

*When people encounter problems, and see me in uniform, they approach me for help. They expect help from me. I have to assist…* (High ranking, 55 years old, separated woman).

Similar views were presented by another interviewee with a view that POs are linked with good leadership of the government:

*Police force is the mirror of the society in the sense that it is something which reflects the Government’s mission in terms of good leadership and that civilians can trust the Government...* (High ranking, 44 years old married man).

Other participants were proud of how they presented themselves in public particularly when they are dressed in their uniforms. They believed that POs’ uniforms display trustful identity in front of the public and obedience of government rules and regulations. In uniform, they felt as a trustful source of information and peace keepers. They stated that civilians comply with regulations in presence of POs who are in uniforms. They said:

*The way I put on uniform is protective. That means, when I walk around and meet people who commit crime, they get worried when they see me ...* (Low ranking, 42 years old unmarried woman)

#### Gender roles in context of Police profession

The participants described various activities as their core functions. They stated that they worked in various environments depending on the officer’s rank, and were involved in multiple tasks within and outside the workplace. Although all participants stated that distribution of duties among POs was not discriminatory, gender difference was somewhat considered in allocation of duties. For instance, women who committed crime were inspected by women officers to avoid the complaint that male officers sexually harass females. Similarly, only males were allocated to guard in the banks because of the perceived high risk of dealing with robbery events. Most participants were of the opinion that few courageous females by nature could work in potentially very risky places. They clarified that distribution of work was done to ensure safety of female officers and also to meet for the needs of women clients.

However, several participants were of different views regarding gender differences in duty distribution in the Police force. Negative notions regarding confidence among female officers emerged because of the practice of allocating risky duties to males and less risky duties to females. They insisted that, gender difference in duty allocation would affect female officers when it comes to leadership and decision making. Men in particular portrayed women as individuals who cannot make quick and independent decisions. They said that quite often women leaders turn to men to look for support before making decisions. However, other men defended women officers as people who are confident, and who can express authority and make decisions just like men:

*Women in the police force are confident… they are confident like soldiers. … The training which women and men receive in the Police College enhances confidence...* (High ranking, 50 years old married man)

#### Gender and leadership

Participants stated that although in the past women were ranked low; currently both men and women have equal opportunities in leadership positions. They insisted that given the professional training which women acquire from the colleges, men perceive women as leaders with sound knowledge. From this perspective, women were perceived as current and future leaders. One participant said:

*Other stations are headed by women. Before I became a station leader here, this station was headed by a woman who made precise decisions just like men do* (High ranking, 54 years old married man)

Most participants argued that it was the mindset of some men that women cannot make firm decisions as leaders. They insisted that both women and men are in the forefront in terms of duty performance and capability to make independent decisions.

#### Gender based nepotism in Police profession

Some participants speculated that female officers were seeking job promotion in exchange of sexual relationship. However, most women opposed the speculation that women were engaging in sexual relationships in order to be promoted. They said:

*No way, perhaps it happened in the past. Currently, there is a selection committee. I used to tell young women* **[**Police officers**]** *that there are no such things* [sexual bribes in job promotion]*…* (High ranking, 54 years old widow)

Most participants said that when it came to job promotion, both men and women were promoted because of hard working and the level of education. Also, they insisted that job promotion was influenced by good performance, and that recognition of a good performer went beyond work station in the sense that even the civilians would be talking about the good performer at a certain station. Consequently, promotion under merits of good performance was transparent. They realized the struggle women made, in order to be promoted, defeated the speculation that they were privileged for promotion in exchange for sex. Also, some senior high-ranking men officers were against the speculation that women were promoted in exchange for sex. They believed that women deserve promotion as a result of their individual efforts:

*“…Personal efforts advance one from one rank to another… Personally, I have never seen that* [sex in exchange for promotion]*; I just hear from the media…”* (High ranking, 50 years old married man).

Others emphasized that job promotion is a matter of working hard and adhering to moral standards:

*If you work hard and trustful according to army moral standards, I think promotion will take place for anybody regardless of whether you are a woman or man* (Low ranking, 42 years old married man).

Another participant complemented:

*No gender issues in job promotion...there are many aspects such as education, job performance, trust and discipline... No sexual bribe* (Low ranking, 44 years old married man).

Overall, most participants opposed the speculations that female POs were promoted to higher ranks in exchange for sex. However, they were aware of a few men and women who were using money to facilitate sexual negotiations, but not for promotion to higher ranks.

#### Dealing with risky sexual temptations

The participants stated that risky sexual practices can be avoided. Thus, they emphasised that sexual desires can be dealt with individually. They said, one needs to be courageous to overcome his/her sexual desires if it happens that a colleague initiates sexual temptations. They said:

*You have to look for activities to help you decrease your sexual desires…sometimes you are advised to exercise, mixing up with other people...* (Low ranking, 32 years old married man)

Others believed that taking part in other activities is an appropriate tool for suppressing sexual desires when one is away from the family:

*If you are tolerant, you can stay longer until you get back to your spouse. When I talk about tolerance, I attach it with multiple meanings. Others may drink and become drunk, others may take part in sports, and both may diminish sexual desires* (High ranking, 54 years old married man).

Also, the participants believed that sexual feelings can be initiated and controlled by individuals themselves. For example, others felt that inviting God in their daily routine will be helpful:

*If you are busy with God issues even the issue of sexual temptations will not be there. When you are done with your job, spend time with your children, bible on your hands reading, keeping your mind busy. You will be safe from sexual temptations* (Low ranking, 38 years married man).

Another participant added:

*Even in the ten Commandments, God stated that, ‘Don’t admire a woman who is not your wife’* (High ranking, 54 years old married man).

Thus, religious beliefs were attached to avoidance of risky sexual behaviour.

They said that under unavoidable sexual temptations, they would use individual tactics to avoid risky sexual practices. For example, they said if a colleague of opposite sex or a client puts them under temptations which may lead to risky sexual practices, they would assertively caution themselves about the implications. One of them said:

*You have to make right decision … you have to ask yourself, if he/she is doing this* [giving money without apparent reasons, lunch or dinner or other kinds of favour] *to me, how many are we?… You must come up with series of questions which will alert you about the implications of accepting offers …* (Low ranking, 32 years old married man).

Women were of the opinion that, although men were sexually greedy, they could trick them. One woman said:

*You are supposed to use your brain. You should be able to recognize sexual temptations. … Truly there are men, who can inflict temptations through free lunch, but it depends on a person* [woman]*, you have to be firm. It is not acceptable to accept all offers…Greediness is the driving force* (Low ranking, 42 years unmarried woman).

Some women recognized that often high-ranking men tended to induce them with sexual temptations. Under such circumstances, they suggested firm professional approaches to handle them:

*You don’t have to become furious when the boss negotiates for sexual relationship ... If he assigns you duty, do it ... Don’t show that you hate him [*Boss*]. He will perceive you differently and give up. He will never entertain those things* [sexual relationships] *any more* (Low ranking, 42 years old unmarried woman)

The participants stated that bosses with risky sexual behaviours often approach different women. They said some women may be trapped because of love and getting favours in various ways. They emphasised that no man will harass women or fire them from work if they refuse to entertain sexual relationships. They commented that sexual temptations at work and outside work place may be controversial. However, it appeared that high ranking officers were proposing sexual relationship to lower ranking women. The senior officers emphasised that it was impossible for women to turn down their request:

*A senior is a senior; you have to agree. He sends you and requests you for something* [sex]*… He tells you he loves you. Now you get confused; you fail to give answers such that if I say, ‘No’ then I will mess up with my job; I will be harassed here!...* (High ranking, 50 years old married man)

Other participants complemented that sexual proposals were approved within the limits of negotiations. However, under unavoidable circumstances, they insisted that protection against HIV infection should be a priority. Whereas some participants cautioned that entertaining love affairs with the boss may distort the working relationship, some supported such relationships provided they do not affect official duties. Thus, sexual favours may be demanded by high ranking officers, but that it does not affect promotion.

#### Emphasis on protected sex

The participants said in case sexual temptations go beyond their control; they would use condoms for protection. They felt that it would be easy to convince their sexual partners to use condoms only if they are in good relationships. They said for example, women may take responsibility of advising their partners to use condoms when they are away from the family. They insisted that under circumstances which may tempt one to engage in sexual relationships outside wedlock, they should wear condoms:

*You may be away from your wife; perhaps attending a seminar the whole month. As a human being it reaches a point where you feel to be with someone…you fall in love with someone and she falls in love with you too… the most important issue here is to use condoms* (High ranking, 50 years old married man)

They expressed trust over usefulness of condoms:

*I trust condom because first it prevents infection, it takes away worries while cheating…it prevents pregnancy and infections if any ... You cannot say that I don’t use, what if the partner cheats without wearing condom?* (Low ranking, 49 years old married man)

Moreover, women supported the idea of condoms use outside marriage relationships. They said:

*At one time, you* [woman] *must sit down and discuss about the current situation* [HIV transmission] *with your partner … he has to use condom; if he carries condom, and not using it, that is his problems. However, majority are keen and would use condoms* (Low ranking, 42 years old unmarried woman)

Another woman added:

*I give high priority to condoms. Nobody should lie you about skipping condom because you must get prepared for anything, you see. Once you use a condom it means you have protection. But if you decide to cheat, you never know your partner’s status* (High ranking, 40 years old married woman).

Thus, both men and women perceived themselves as good advocators of condom use in the institutions they worked for and the general community.

Some participants stated that support from a spouse would be a motivation to use condom outside wedlock. One participant said:

*At one time you* [woman] *must sit down and discuss about the current situation [HIV transmission] with your spouse … you have to use condom. If he carries condom, and not using it, it is his problems. However, majority are keen and would use condoms* (Low ranking, 42 years unmarried woman)

Others added that a dialogue and preconscious mindset can facilitate one to use a condom. Participants argued that if a sexual partner does not support condom use, unprotected sexual intercourse should not be entertained. They suspected that anyone who initiates to have sex without wearing a condom may be infected and wanting to spread the infection to others:

*If she does not like it* [condoms] *then I know she has her own reasons; she may be infected with HIV wanting to die with me and that is why she is insisting on that thing* [sex]*… one may tell you that she has her own condom and would prefer not to wear yours. Perhaps her condom has worn out. While in darkness or drunk, you end up using her broken condom…therefore, it is better not to accept using her condom* (Low ranking, 42 years married man).

Overall, good relationships between sexual partners and frequent reminders emerged as facilitators towards condom use.

#### Unsure of condom use among sexual partners

According to religious beliefs, some participants insisted that both sexual practices outside wedlock as well as condom use are immoral. For instance, they said that the Roman Catholics believe that condoms were made before HIV, and therefore they were not meant for HIV prevention. One participant said:

*I am a Roman Catholic. We Catholics are not allowed to use condoms, not because of extra marital sexual partners … Therefore, the best medicine is abstinence …* (Low ranking, 42 years old married man)

In addition, some men were uncertain about encouraging condom use with either casual sexual partners or their spouses. Under normal circumstances, they believed that entertaining condom use in marriage may facilitate mistrust between couples:

*When you start giving directives to use condoms, already you are inducing temptations to practice sex outside wedlock. That is something that I shall never entertain between my wife and I. Carrying condoms in my pocket will depict cheating…* (High ranking, 50 years old married man)

Another participant added:

*When you tell someone* [sexual partner] *to use condoms when she/he goes out, that means you are encouraging / telling him/her to cheat…* (Low ranking, 49 years old married man)

Similarly, other participants found it difficult to involve their sexual partners in matters related to condoms use. They believed in the notion that once they ask a partner to carry condom it means granting permission to practice sex with other sexual partners when they have sex outside their wedlock. They said:

*That will be very difficult. Let us say I am married, and then advising my husband to carry condoms and use in case he fails to tolerate sexual feelings while on safari* [journey]*, it is not easy. ... it is difficult to motivate your partner to carry condoms...perhaps he didn’t plan to have sex, but once you initiate he will start and you cannot blame him* (Low ranking, 24 years old married woman)

Others were not in favour of condom use because of their perceptions about the uses of condoms:

*I have used them* [condoms] *a lot, I got tired when I finish ejaculating. Yes, you feel tired; this has a meaning... I wondered why. Perhaps as I ejaculate some sperms remain and those are the ones which make me tired. I use glycerine* [lubricant] *instead to avoid friction... no bruises because you apply glycerine. You finish* [ejaculate] *fast than when you use condoms* (High ranking, 44 years old married man).

Also, the issue of trusting condoms from abroad appeared to discourage some people to their use. This led to some participants to believe that condom use fuels HIV transmission because they are packed with the virus. They said:

*Perhaps condoms are implanted with strange things* [HIV] *because instead of decreasing HIV transmission, they increase the infection rate; everybody understand what condoms are for but we are surprised why HIV keeps on increasing while condoms are being used?* (High ranking, 50 years old married man).

Another participant shared similar sentiments that condoms have HIV and went further to express that it is better to use lubricants than using condoms. He said:

*It is not a secret...you know there is this notion that condoms were imported from abroad and they have HIV... Therefore, people have stopped using them completely... Since the aim of condom use is to decrease friction, I use lubricants instead* (High ranking, 44 years old married man).

Whereas some participants claimed that condoms decrease sexual pleasure, others differed in opinions. They challenged the notion that condoms decrease sexual pleasure. Instead, they clarified that extra marital sexual practice insinuates feelings of cheating; and it is the feeling of cheating which decreases sexual pleasure:

*The main problem here is not that condom decreases sexual pleasure. This is an individual’s perception while engaging in extramarital sexual affairs. The cheating itself decreases sexual pleasure. When this happens, the man may think of a condom as a source. This is his perception; the perception that leads to anxiety which emanates from cheating… ‘Maybe I will get diseases; how will it be?’…* (Low ranking, 42 years old married man).

Another participant commented on the same issue of condom use and sexual pleasure:

*It is true. Even I thought of the same* [condoms decrease sexual pleasure]. *Our organs* [sexual organs] *do not meet. Now if they don’t meet, one will… I don’t know about women. Perhaps, they enjoy. I cannot give a straight answer to that… diseases are many. When you consider our responsibilities, it is better to protect ourselves from diseases* (High ranking, 54 years old married man)

One woman complemented on the issue of condoms and sexual pleasure by saying that:

*Those are people’s perception* [that condoms decrease sexual pleasure] *because pleasure is always there… Personally, I still experience sexual pleasure when I use a condom, and my sexual desires are met…* (Low ranking, 42 years unmarried woman)

On the contrary, the participants recalled consequences of low condom use such as HIV transmission and unintended pregnancies. Both men and women perceived those who practice unprotected sexual intercourse outside marriage as people who do not understand that they are in the era of HIV prevention. They condemned them as people who want to strangulate themselves. They emphasized that unprotected sexual intercourse outside wedlock must be avoided in the sense that one should avoid getting HIV infection. Some of them felt that unprotected sexual intercourse outside wedlock is dangerous because people are not sure about the status of the partner.

*When people go outside wedlock without using condoms…It contributes a lot in HIV transmission. HIV infections will increase among couples than in the general population...* (Low ranking, 24 years old married woman)

They said that any sexual relationship outside wedlock should be viewed as a chance of getting HIV infection. Also, both men and women portrayed pregnancy outside wedlock as immoral consequence.

### CATEGORT 2: Perceived readiness to enrol in early HIV vaccine trials among POs

#### Partner involvement in HIV vaccine trials

Most participants shared their positive views about participation in HIV vaccine trials. They demonstrated understanding of safety issues when it comes to participation in HIV vaccine studies as one of participants attested that “*It is not true that researchers implant harmful things* [HIV vaccines] *in people*...” (High ranking, 50 years married man)

In the same way, other participants said that conducting research implies efforts to search for good things through scientific evidence. They opposed the notion that researchers are not trustful people, and that they use research money for personal benefits. One of participants reported that “*They* [researchers] *are good because they search for benefit of the majority; not for themselves ...”* (High ranking, 54 years old man).

Several participants portrayed the advantages of HIV vaccine studies. For instance, they stated that it would be compulsory to participate in HIV vaccine trials because vaccines in the trials are not infectious. Also, they understood that the aim of volunteering in the vaccine trials is to save people. They gave examples of elimination of measles, diphtheria and polio through vaccines. Thus, they insisted that it is important to participate in HIV research studies without giving up. One participant said:

*We have to be courageous that this vaccine will help in future* (High ranking, 50 years married man).

*I would volunteer because that vaccine will be useful to me as well as to my fellow Tanzanians* (Low ranking, 24 years married woman)

In addition, most would allow their spouses to take part in HIV vaccine trials. They said they would clarify trial concepts to ensure understanding among spouses. One participant said:

*Yes, I would convince my sexual partner to take part because she is my lover. If she agrees both of us will be safe* (High ranking, 54 years old married man).

However, they emphasised that voluntarism should be the basis of taking part in the sense that spouses should have freedom to make independent decision after understanding the trial concepts. They said individual decision is important when it comes to participation in HIV vaccine trials:

*It is supposed to be self motivation. If you decide to do something then do it from your heart; don’t listen to people’s words*... (Low ranking, 32 years old married man)

#### Obstacles to condom use in phase I/II HIV vaccine trials

The participants were unsure of researchers’ intention in relation to a requirement to avoid pregnancy during the period of being enrolled in a HIV Vaccine trial including using condoms in sex. Some participants preferred not to take part in the HIV vaccine trials together with spouses so that if the vaccine became ineffective one of the spouses would be saved. They suspected that the researchers did not know the side effects of the HIV vaccine. One woman said:

*They* [researchers] *are not sure of themselves if they will succeed or if it will cause side effects...* (Low ranking, 24 years old married woman).

Also she felt that it would be very difficult to convince the husband.

*I am not sure if he will be convinced to take part in the trial* (Low ranking, 24 years old married woman)

They expressed lack of comprehensive education among spouses particularly on the scenario of using condoms during HIV vaccine trials. They felt let down especially if the spouses [wives] will express dissatisfactions in sexual relationships because of using condom. Culturally, they envisioned shame if the wives will inform the mother in law about sexual dissatisfaction. They perceived that it will be very difficult to convince their spouse to use condoms during the trial but in extramarital relationships it is acceptable. They emphasised that in extra marital relationships, it will be possible to use condoms. One man said:

*You* [man] *can tell her* [woman who is not the spouse] *that I am married; therefore I* [man] *cannot practice sexual intercourse with you* [woman who is not the spouse] *without a condom* (Low ranking, 32 years old married man).

They felt that although condom use during the vaccine trials will be meant for self-protection and partners’ safety, it may contribute to break ups of relationship. Therefore, emphasis on avoidance of unprotected sexual intercourse was crucial.

Several participants believed that participation in preventive HIV vaccine trials may endanger their lives. They were hesitant to take part in the vaccine trials because they were uncertain of getting support in case of side effects from the vaccine. They stated:

*Perhaps it may bring side effects in future...* (Low ranking, 43 years old married woman).

They believed that one may be vaccinated and after one year becomes infected without having health insurance. Overall, the findings underscore both men and women’s views in the light of the research questions.

## Discussion

This discussion is loosely guided by gender differences, sexual relationships and HIV prevention efforts. Both men and women in the Police force perceived themselves as role models. Gender power difference in roles distribution, and sexual relationships emerged as important issues amongst POs. Under normal circumstances, avoidance of risky sexual temptations appeared crucial to prevent untoward health outcomes. Risky sexual practices were considered avoidable, and condoms use seemed applicable when partners were in good relationships. However, individual preferences appeared to hamper condoms use. Participation in HIV vaccine trails was perceived as a good thing, yet researchers were not trusted for they were suspected that they could implant harmful things in participants’ bodies which was obviously not true according to international medical ethical norms.

### Self perception, sexual relationship and gender difference

The POs’ self-perception that they are the mirror of society implies that their duties and behaviours are geared towards shining out in the society. Thus, adhering to moral standards appears to promote not only respect, but adherence to safer life style. In a similar vein, police officers are expected to uphold safe sexual practices as part of discipline. Nevertheless, the practice of having extra marital sexual relationships and the possibility of low condom use is not surprising in uniformed forces. In a previous study, among POs in Tanzania, only 54% used condoms during extra marital sexual practices [5]. Also, some of the participants [Police officers] who enrolled and completed phase I/II HIV vaccine trials reported to engage in unprotected sexual practices with a partner of unknown HIV status [10] which refutes discipline in sexual practices among all officers. Elsewhere, military personnel with respect to sexual activities, on the average had more than one sexual encounter without condoms use implying that condoms use was low [11, 12]. Similarly, in Caribbean Military Personnel, more than 60% who reported condom use during last encounter with commercial sex workers also reported engagement in unprotected sex sometimes [13]. In the Botswana Defense Force, more than half of studied participants reported having one casual partner and one regular partner, and only 51% reported always using a condom [14].

### Sexual behaviour and gender differences

The fact that participants viewed sexual feelings as human nature, yet felt that they could control such feelings implies that self discipline is crucial in dealing with sexual temptations. In view of daily circumstances, sexual temptations may persist among uniformed forces. This study indicates individual assertiveness in dealing with risky sexual behaviours implying that people can fight against HIV transmission by using self interventions. Women being the most affected by HIV/AIDS in the Police force and general population in Tanzania and sub-Sahara Africa indicate if empowered they can contribute a lot against HIV transmission through discouraging risky sexual temptations from men. Men in uniformed forces need to increase their understanding of the vulnerability of women to HIV infection. Lack of seriousness on gender sensitivity by the rank of male officers can increase the risk of sexual abuse for women. In South Africa, efforts against HIV infection among women was compromised by gender power dynamics [15]. In Tanzania, young female POs lamented how some high ranking officers were trying to bribe them for sexual relationships [7]. In the light of the current findings, such men can be controlled by assertive responses from women. Thus, empowerment of women through increasing their self-esteem and negotiating power can impact on the behavior of men eager for sex. Also female autonomy can significantly decrease the risk of HIV infection by having one partner who has only one sexual partner [16]. A previous study suggests that tackling HIV in the military personnel requires a rigorous examination of social factors, gender issues included [17]. While the current findings indicate that both men and women behave differently when it comes to sexual relationships, it is evident that both are aware of the consequences. As risky sexual practices persist in uniformed forces, peer educator intervention has been used, but unfortunately it does not appear to capitalize ahead of interpersonal relationships [18].

### HIV prevention gender perspectives

It has been recognized that uniformed services are increasingly an at-risk population for HIV/AIDS [19–21]. Despite wide and long-term promotion on use of condoms, persistent negative attitudes towards condoms may hinder their use partly due to gender differences. In this study, several men and women supported use of condoms; however, some were claiming that condoms decrease sexual pleasure. Similarly, in the Botswana Defence Force, one of the reasons for not using condoms is that condoms make sex less enjoyable [14]. Negotiation to use condoms between sexual partners is not new in the era of HIV/AIDS. In a study from Botswana among military personnel, most participants indicated that it is alright for women and men to ask their spouses to use condoms, and that by using condom it shows one cares for his/her sexual partner [14]. It is anticipated that if women become more confident in their relationships with their sexual partners, the chances that they will be able to protect themselves and their families from infection with HIV increase. Women can develop skills for talking with their partners and negotiating condoms use. In some settings, wives of men in uniform have been encouraged to discuss condom use by their husbands in sexual relations outside the marriage [22]. Thus, individual efforts in negotiating condoms’ use may play a big role in HIV prevention. It is evident that correct and consistent condom use is highly effective as evidenced through decrease in HIV transmission [1].

The Tanzanian Government is consistently sensitizing people on proper use of condoms in the context of diseases prevention including HIV. However, the current findings demonstrate that consistency in use of condoms outside wedlock is controversial. People may perceive and act differently when it comes to sexual practices. A previous study among military personnel revealed that the number of sexual partners was significantly associated with condom use attitude and behavior. The majority of the participants (61.8%) indicated that they had sex with multiple partners in the past three months, and 27.5% of them having negative attitudes towards condom use [11]. In Ethiopia, the overall prevalence of consistent condom use among Western command was very low [23].

Participation in Phase I/ II HIV vaccine trials require people to be at low risk of acquiring HIV infection, which requires an understanding between sexual partners that both of them protect themselves against HIV acquisition and pregnancy during the trial is crucial. The fact that men demonstrate readiness to motivate their spouses to take part in HIV vaccine trials is worth noting. On the contrary, a previous study within the same Police cohort showed that spouses were against partner participation in HIV vaccine trials in the sense that the experimental vaccine could affect them in diverse ways [8]. Mistrust of HIV vaccine trials is not unique in this study, implying that more education on the concepts and conduct of HIV vaccine trials is needed [24–26].

### Limitations

This study had a number of limitations. The findings cannot be generalized to the whole population of POs in Dar es Salaam as the present study focused specifically on POs who were participating in HIV vaccine trials. Accordingly, the recruited participants for the study were from a group of POs who had been exposed to information sessions [Barazas] on HIV/AIDS and HIV preventive vaccine development which might have influenced their responses in one way or another. The age of study participants might not reflect the sentiments of younger Police officers; however, they had the benefit of a lot of experience in terms of HIV/AIDS knowledge. Overall, the findings add value in the response to HIV epidemic in terms of addressing behavioural contexts and HIV infection as well as participation in HIV vaccine trials from the perspective of a uniformed force population. Also, information in this paper may be useful in future HIV vaccine trials in Tanzania and elsewhere.

## Conclusions

This qualitative study was part of a follow up of a large HIV/AIDS study among POs done in Dar es Salaam, Tanzania. In particular, this study was intended to expand our understanding of how individual POs perceived their roles in line with HIV prevention. The findings increase understanding of context specific efforts towards HIV prevention, and that POs are struggling to avoid risky sexual practices regardless of gender. The observed uncertainties in promoting condom use underscore the importance of condoms promotion. The intention to participate in HIV vaccine trials by POs towards development of an effective vaccine is encouraging.

## Abbreviations

AIDS: Acquired Immune Deficiency Syndrome
HIV: Human Immune Deficiency Virus
POs: Police Officers
STIs: Sexually Transmitted Infections
UNAIDS: Joint United Nations Programme on HIV/AIDS
WHO: World Health Organization
